# Neuroprotective Effects of Kinin B2 Receptor in Organotypic Hippocampal Cultures of Middle-Aged Mice

**DOI:** 10.3389/fnagi.2019.00168

**Published:** 2019-07-12

**Authors:** Mariana Toricelli, Sebastiana Ribeiro Evangelista, Larissa Rolim Oliveira, Tania Araujo Viel, Hudson Sousa Buck

**Affiliations:** ^1^Department of Physiological Sciences, Santa Casa de São Paulo School of Medical Sciences, São Paulo, Brazil; ^2^Research Group on Neuropharmacology of Aging—ReGNA, São Paulo, Brazil; ^3^School of Arts, Sciences and Humanities, University of São Paulo, São Paulo, Brazil

**Keywords:** organotypic culture, neuroinflammation, bradykinin, kallikrein-kinin system, Alzheimer’s disease

## Abstract

Aging is a multifactorial phenomenon that results in several changes at cellular and molecular levels and is considered the main risk factor for some neurodegenerative diseases. Several evidence show the participation of the kallikrein-kinin system (KKS) in neurodegeneration and this system has been associated with inflammation and immunogenic responses in the central and peripheral systems by the activation of the B1 and B2 receptors. Previous work by our group showed that bradykinin (BK) and the B2 receptor played a possible role in neuroprotection. Therefore, the objective of this study was to evaluate the participation of B2 receptors in cell viability, neuroinflammatory response and neuroplasticity in organotypic hippocampal cultures (OHCs) of 6- and 12-month-old mice. It was observed that activation of the B2 receptor by bradykinin decreased the inflammatory response and increased plasticity in 12-month-old slices. Conversely, there was an increase in the inflammatory response and a decrease in neural plasticity in the 6-month-old slices. In both ages, an increase in cell viability was observed. This data suggests that the function of the kinin B2 receptor in the hippocampus is modulated by age, providing neuroprotective action in old age.

## Introduction

It is a fact that the global population has been passing through a significant demographic transition. Many societies are no longer predominantly formed by young people and adults; they are turning into societies composed of a larger proportion of older people (Custodio et al., 2017). Although this data demonstrates an improvement in life expectancy, it also points to the possibility of an alarming increase in the number of people affected by diseases related to aging, such as Alzheimer’s disease (AD; Wimo et al., 2017).

The aging process is associated with a progressive decline in responsiveness and ability to adapt to environmental challenges. This decline in functions includes deficits in the central nervous system (CNS), with structural and biochemical alterations, repair deficiency, the formation of senile plaques and neurofibrillary tangles, which leads to macroscopic and microscopic changes culminating in neuronal death (Custodio et al., 2017).

Although there is scientific advances and increased knowledge in the neurological field, the development of new drugs to treat neurodegenerative diseases continues to be a challenge. Greater knowledge about the neurodegenerative process is essential for the discovery of new molecules with therapeutic and diagnostic potential.

Evidence has emerged showing the involvement of the kallikrein-kinin system (KKS) in neurodegenerative diseases. Nitsch et al. (1998) observed that bradykinin (BK) receptors can modulate the proteolytic process of the amyloid precursor protein (APP). Cultured cells treated with bradykinin increased β-APP secretion, and this result was reversed using antagonists specific for BK receptors (Nitsch et al., 1998).

Another study found that a single dose of BK applied to the rat hippocampus promotes Tau protein hyperphosphorylation, leading to decreased learning and memorization in rats (Wang and Wang, 2002).

In previous studies conducted by our group using knockout mice for the B1 or B2 receptors (KOB1 or KOB2, respectively), we investigated the role of the kinin receptors in learning and memory processes in aging. It was observed that in 12-month-old KOB1 mice memory was preserved when compared to C57Bl/6 animals, however, age-matched KOB2 animals did not show the same performance in behavioral tests (Lemos et al., 2010). It was also suggested that kinin B1 receptor (BKB1R) participated in the impairment of short-term memory in traumatic brain injury in rats (Dong-Creste et al., 2016), while kinin B2 receptor (BKB2R) played a possible neuroprotective role in AD. Chronic infusion of Aβ peptide in KOB2 led to accelerated cognitive deficit and increased AB deposition when compared to KOB1 mice. KOB1 mice also showed increased densities of BKB2R after Aβ peptide chronic infusion (Amaral et al., 2010; Caetano et al., 2015).

These data reinforce the involvement of the KKS in AD, but it is not possible to say if the behavioral changes and the increase in amyloid plaques are a cause or a consequence of the association with the inflammatory process and neuronal loss due to the presence of Aβ deposits.

Our hypothesis is that BK, through the activation of the BKB2R, could elicit different responses in the CNS, depending on inflammatory state of the brain. Considering that an increase in inflammation is observed during the aging process (Franceschi and Campisi, 2014), the aim of this study was to evaluate the participation of the BKB2R in cell viability and inflammatory response in organotypic hippocampal cultures (OHCs) from mature adult and middle-aged mice (6 and 12 months old, respectively). The understanding of different temporal effects of BK in the hippocampus could be relevant for new therapies development concerning neurodegenerative diseases.

## Materials and Methdos

### Animals

Male 6- and 12-month-old C57Bl/6 mice (number of technical and biological replicates is indicated in figure legends) were obtained from the FCMSCSP Animal Facilities, São Paulo, Brazil. The animals were kept in a light-dark cycle of 12/12 h, in temperature and humidity controlled environment, with water and food *ad libitum*, in mini-isolators placed in ventilated racks (Alesco, Brazil).

Mature adult mice (6 months old) were used as a control group since, at that age, their behavior is similar to younger animals’ behavior. Middle-aged mice (12 months old) show detectable senescent changes but not all biomarkers of aging. The old animal group (at least 18 months old; Flurkey et al., 2007) was not included in this study since the quality of OHCs from animals older than 12 months old was not consistent (personal observation).

All efforts were made to follow the 3Rs (Replacement, Reduction, and Refinement) to minimize the animal number used and their suffering. The experimental proceedings were performed according to the ethics principles for the use of laboratory animals of the Brazilian Society of Laboratory Animal Science (SBCAL, Brazil). The experimental protocols were approved by the Animal Ethics Committee from Santa Casa de São Paulo School of Medical Sciences, No. 001/14.

### Preparation of Organotypic Cultures

Animals were anesthetized and killed by decapitation and their brains were rapidly removed and kept in cold 25 mM HBSS buffer, pH 7.4, containing 1 mM Penicillin/Streptomycin (dissection buffer). From this point, until plating, all equipment and tissue were kept in cold ice. The caudal part of the brain was glued onto a vibratome stage (Leica VT1000S) and flooded with dissection buffer. Under aseptic conditions, 300 μm sagittal slices (6–9 per brain) were taken. For this, the cutting blade was positioned at an angle of 15°, cutting speed 3.8 and a frequency of 10 and the hippocampus was dissected out using sterile tweezers. The dissected hippocampal slices were then transferred to a six-well culture dishes using a sterile 3 ml plastic pipette modified to widen the opening. Four to six slices were placed onto sterile 0.4 μm pore membrane inserts (Millipore PICM03050). During the first week a nutrient enriched culture medium was used for the complete recovery of the hippocampal slices containing: 50% MEM with Glutamax-1 (Gibco: 42360-032), 10% HAM’S F-10 Nutrient mix (Gibco: 12390035), 1 mM GlutaMAX^TM^ Supplement (Gibco: 35050061), 1 mM Sodium Pyruvate (Gibco: 1360070), 25% heat-inactivated horse serum (Gibco: 26050-070), 25% HBSS (Gibco: 14025-092), 37 mM D-Glucose (Sigma: G8270), 1% Antibiotic/Antimycotic (Gibco: 15240-062). After this period, the inserts were kept in 1 ml of maintenance medium 50% MEM with Glutamax-1 (Gibco: 42360-032), 25% heat-inactivated horse serum (Gibco: 26050-070), 25% HBSS (Gibco: 14025-092), 37 mM D-Glucose (Sigma: G8270), 1% Antibiotic/Antimycotic (Gibco: 15240-062). Dishes were maintained in incubators at 37°C, 5% CO_2_ for up to 4 weeks. The medium was changed three times a week.

### Drug Treatment

After 1 week of stabilization, OHCs from 6-month-old (6-OHC) and 12-month-old (12-OHC) animals were treated with 300 pM of BK or 300 pM of BK + 200 pM of B1 antagonist (BK+AntB1) [AntB1: (desArg10)-Hoe140] [3-4hydroxyphenyl-propionyl-des-Arg9-D_Arg (Hyp3, Thi5, D-TiC7, Oic8)_BK], to block the possible effects of desArg9BK (Wirth et al., 1991; Dong-Creste et al., 2016), added to 1 mL of maintenance culture medium, all purchased from Sigma^®^, and were treated for 30 days. Every 3 days the culture medium containing the treatments was replaced. The BK dose used, 300 pmol, was determined based on data from the literature (Noda et al., 2007) and considering unpredictable actions of the peptidases activity in heated serum and in the brain tissue. The addition of [desArg10]-Hoe140 was performed considering the possible activity of tissue carboxypeptidase M-like enzymes converting BK into the B1R agonist desArg9-BK (Dong-Creste et al., 2016; Haddad and Couture, 2017).

### Propidium Iodide Staining

At the end of treatments, culture medium was reserved for further analysis. Hippocampal slices were washed with 1 mL PBS and 1 μg/mL propidium iodide was added to each culture well. The slices were incubated for 15 min in the dark. After washing with PBS, images of slices were acquired using a ZOE^TM^ Fluorescent Cell Imager (Bio-Rad Laboratories) and analyzed using ImageJ software.

### Western Blotting

The densities of synaptophysin, B1 and B2 receptors and NeuN, were evaluated by western blotting with the use of specific antibodies. Briefly, the hippocampal tissues from each insert were collected and homogenized in cell lysis buffer (Cell Lysis Buffer [10X] #9803, [1X], ph7.5, Cell Signaling Technology^®^) and protein extracts were quantified using the Bradford method (Bio-Rad Protein Assay Dye, Bio-Rad). Equal amounts of protein from each sample were separated by polyacrylamide gel electrophoresis and then transferred to a polyvinylidene fluoride membrane (PVDF). The membrane was blocked, incubated with the specific antibodies and then with peroxidase-conjugated antibodies. The membranes were developed with the use of a chemiluminescence detection reagent (SuperSignal^®^ West Pico Chemoluminescent Substrate, Thermo Scientific) and the bands were analyzed and quantified using ImageJ software. The primary antibodies used were: synaptophysin (1:5,000—Abcam), B1 (1:1,000—Alomone labs) and B2 receptor (1:1,000—Alomone labs) and NeuN (1:1,000—Millipore). Map2 was used as the inner standard (1:2,000—Abcam).

### Evaluation of Neuroinflammatory Response From Bradykinin Treatment

The levels of interleukin 1α and 6, MCP-1/JE/CCL2, MIP-1B/CCL4 were evaluated in the culture medium using a multiplex system, according to the manufacturer’s instruction. These markers were quantified by Magnetic Luminex^®^ Assay—Mouse Premixed Multi-Analyte Kit R&D systems.

### S100b Analysis

The S100b level released by hippocampal slices was analyzed in the medium by Mouse S100b (S100 Calcium Binding Protein B) Elisa Kit (Elabscience) according to the manufacturer’s instruction.

### BDNF Analysis

The level of brain-derived neurotrophic factor (BDNF) in culture medium was analyzed by Quantikine Elisa Total BDNF Immunoassay^®^ (R&D systems) according to the manufacturer’s instruction.

### Statistical Analysis

The results were expressed as means ± SEM. Data were analyzed using one-way ANOVA followed by Bonferroni’s multiple comparisons test for each brain area. The results were organized into a database using the GraphPad Prism 5 statistical program (GraphPad Software Inc., San Diego, CA, USA). Only probability values (P) less than 0.05 were considered statistically significant. Data was generated from technical and biological replicates where the means from technical replications of the different biological samples were used for the statistical analysis.

## Results

### Morphological Characterization of Slice Cultures

**Figure 1:**
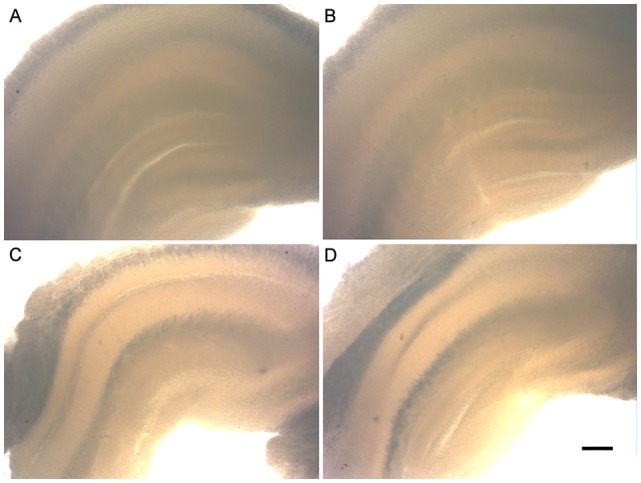
Morphology of the organotypic hippocampal slice cultures of 6- and 12-month-old animals (6-OHC and 12-OHC). Image shows the well preserved tissue architecture of the hippocampus and the absence of black spots, characteristic of cell death. **(A)** 6-OHC, 7 days *in vitro* (DIV). **(B)** 6-OHC, 30 DIV. **(C)** 12-OHC, 7 DIV. **(D)** 12-OHC, 30 DIV. Scale: 100 μM.

Unlike most research groups that use animals up to 10 days old to set hippocampal organotypic cultures, whose young neurons have greater plasticity and resistance to survive under culture conditions, in the present study the hippocampal slices were from 6-and 12-month-old animals. Figure 1 shows the morphology of the hippocampal slices of the C57Bl/6 animals at 6 and 12 months of age after being cultured for 30 days *in vitro* (30 DIV). It is important to note the preserved tissue architecture over the weeks. All areas are well defined (Dentate Gyrus, CA1, CA2 and CA3) and did not present black spots, characteristic of cell death.

### Hippocampal Slices From Old Animals Had Low Rates of Cell Death

**Figure 2:**
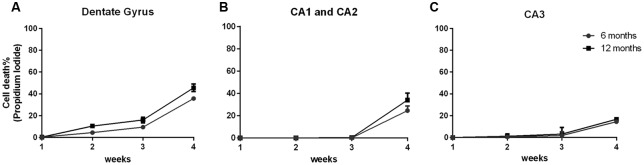
Hippocampal slices of old animals remain viable over 4 weeks. Percentage of cell death along 4 weeks of six hippocampal slices of three C57Bl/6 animals at 6 and 12 months old, stained with 1 μg/mL propidium iodide. **(A)** Dentate Gyrus area; **(B)** CA1 and CA2 areas; **(C)** CA3 area.

In order to demonstrate that the cell viability of the 6- and 12-OHC were maintained throughout the experimental time-points, hippocampal slices were collected once a week and stained with propidium iodide. All hippocampus areas showed low cell mortality up to 3 weeks in culture (Figures 2A–C).

After 4 weeks of maintenance, all hippocampal areas exhibited cell death below 50%. Therefore, this *in vitro* tool showed itself to be an important and useful method to obtain data similar to those found *in vivo* using a decreased number of animals.

### Treatment With Bradykinin Increases Cell Viability

**Figure 3:**
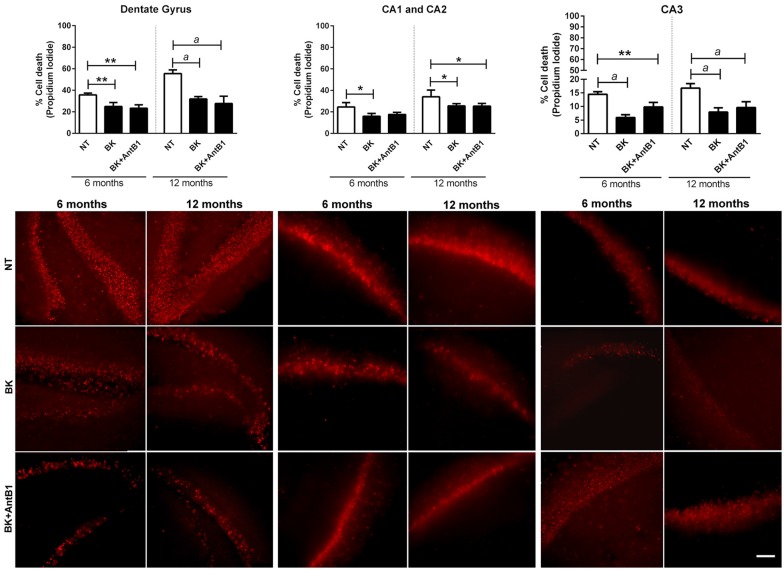
Treatment with BK and BK+B1 antagonist preserved different hippocampal areas from cell death. Image shows quantification of cell death and representative histological images of cell death (in red) of hippocampal slices of 6- and 12-OHC treated with 300 pM of BK or 300 pM of BK+200 pM of B1 antagonist (AntB1) for 30 DIV. NT, non-treated slices. Histograms and vertical bars are means ± SEM. *p < 0.05, **p < 0.01, ^a^*p* < 0.0001, analyzed with one-way ANOVA followed by Bonferroni’s multiple comparisons test. Technical quadruplicate of four animals was used for each treatment. Scale bar is 100 μm.

Our group’s previous work highlights a possible BKB2R role in memory maintenance and neuroprotection (Lemos et al., 2010; Caetano et al., 2015). To explore these results further, hippocampal slices from young and adult animals were treatment with BK or BK+AntB1 and cell death was evaluated after 30 DIV of treatment. In the dentate gyrus, where there is greater cellular loss compared to the other hippocampal areas, BK significantly reduced cell death in both the 6- and 12-OHC (30.1%, *p* < 0.01 and 42.5%, *p* < 0.001, respectively, Figure 3). It is interesting to note that no greater protection was observed after adding AntB1, indicating that BK through the BKB2R was the effective neuroprotective agent. In the CA1 and CA2 areas, treatment with BK also resulted in a significant decrease of 35.0% (*p* < 0.05) in cell death of 6-OHC. However, the co-treatment with AntB1 did not promote a statistically significant effect. In the12-OHC, where cell loss is greater, BK promoted a significant reduction of 24.9% (*p* < 0.05) in cell death, and this was maintained when AntB1 was added to the culture medium (Figure 3). In the CA3 hippocampal area, the same protection was observed, as BK promoted a significant reduction in cell death of 59.3% in 6-OHC (*p* < 0.0001) and 57.3% in 12-OHC (*p* < 0.0001). The co-treatment with BK and AntB1 maintained the neuroprotection in both 6- (32.2%, *p* < 0.01) and 12-OHC (52.8%, *p* < 0.0001; Figure 3).

### Bradykinin Treatment Changes Neuronal Marker Levels

Protein expression of MAP2, NeuN, synaptophysin and B1 and B2 receptors in the 6- and 12-OHC were evaluated after 30 DIV treatment.

**Figure 4:**
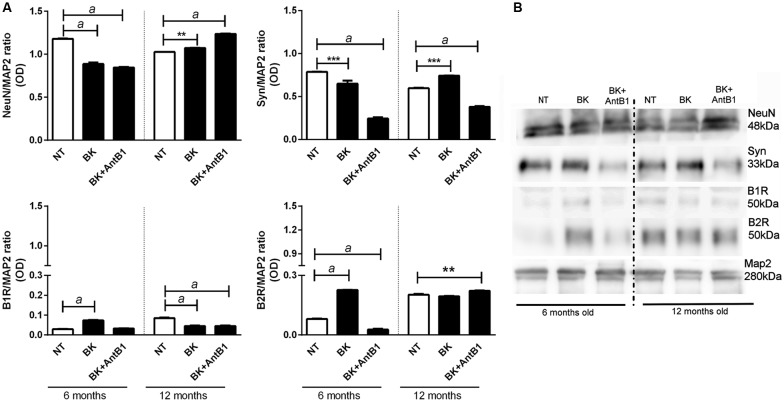
Analysis of neuronal markers and B1 and B2 receptor densities in the hippocampal slices of 6 and 12 month old animals.** (A)** Relative optical density between NeuN, synaptophysin, BKB1R, BKB2R and MAP2 (internal control). **(B)** The Western blot assay was performed to analyze the expression of the following proteins: NeuN, synaptophysin, B1 receptor and B2 receptor. Hippocampal slices of 6- and 12-month-old C57Bl/6 animals treated with 300 pM BK or300 pM BK + 200 pM B1 antagonist (AntB1) for 30 DIV. OD, optical density; NT, non-treated slices. Histograms and vertical bars show the means ± SEM. **p < 0.01, ***p < 0.001, ^a^*p* < 0.0001, analyzed with one-way ANOVA followed by Bonferroni’s multiple comparisons test. Technical triplicate of three animals was used for each treatment.

In the samples from 6-month-old mice, incubation with BK or BK+AntB1 significantly decreased the density of NeuN in hippocampal cells (by 24.8% and 71.6%, respectively, *p* < 0.0001) suggesting a deleterious effect. However, in the 12-month-old samples, the effect was the opposite, as BK and BK+AntB1 significantly increased the neuronal density 1.0-fold (*p* < 0.01) and 1.2-fold (*p* < 0.0001), suggesting a neuroprotective effect (Figures 4A,B).

Concerning the density of synaptophysin, there was a significant decrease of 17.5% (*p* < 0.001) in the levels of synaptophysin in hippocampal samples from 6-month-old mice treated with BK alone and a decrease of 31.1% (*p* < 0.0001) in those treated with BK +AntB1. However in adult animals (12 months old) BK treatment significantly increased the levels of synaptophysin (1.25-fold, *p* < 0.001) indicating that bradykinin plays an important role in mature animals, protecting neurotransmission. When the B1 antagonist was added, the levels of synaptophysin dropped dramatically (48.9%, *p* < 0.0001) suggesting that BKB1R may play a role on the quality of synapses in adult animals (Figures 4A,B). These data indicate that the KKS acts differently in relation to the age of the animals, having a beneficial role only in adult mice.

In the evaluation of the expression of B1 and B2 receptors, a modulation was observed throughout the treatment period. As previously reported, both B1 and B2 receptors were detected in untreated samples of young mice (Viel et al., 2008). Treatment with BK alone significantly increased B1 receptors (by 2.5-fold, *p* < 0.0001) in the 6-months-old samples, but significantly decreased (47.0%, *p* < 0.0001) the density of this receptor in the 12-month-old samples (Figures 4A,B). The addition of BK + AntB1 made no difference in the 6-month-old samples but maintained the decreased density of B1 receptor in 12-month-old samples (47.0%, *p* < 0.0001). Concerning the B2 receptor, treatment with BK significantly increased its density in 6-months-old hippocampus slides (by 3.5-fold, *p* < 0.0001) and the combination with AntB1 promoted a dramatic decrease of this receptor (65.8%, *p* < 0.0001). In the 12-month-old samples, only BK + AntB1 promoted a significant increase in B2 receptor density (1.1-fold, *p* < 0.01; Figures 4A,B). These data reinforce the theory that the KKS responds in different ways according to the age of the animals.

### Change in the Level of Cytokine Expression Following Bradykinin Treatment

Kininergic receptors are widely distributed throughout the CNS of various mammals. They are involved in the modulation and orientation of microglia functions and the neuroinflammatory process, important in neurodegenerative diseases (Couture and Lindsey, 2000). To analyze the relationship between KKS and inflammation in our model we quantified the level of cytokines released into the culture medium after treatment with BK or BK+B1 antagonist.

There was a marked increase in the release of IL-1α in samples from 6-month-old mice following BK treatment (3.2-fold, *p* < 0.0001), suggesting its potential inflammatory action. Interestingly, when AntB1 was added to the media, the density of IL-1α did not change, showing that the inflammatory effect observed after BK administration was dependent on the B1 receptor. However, in samples from adult animals, the treatments resulted in a decrease in IL-1α release, thus indicating the neuroprotective potential of kallikrein kinins system.

**Figure 5:**
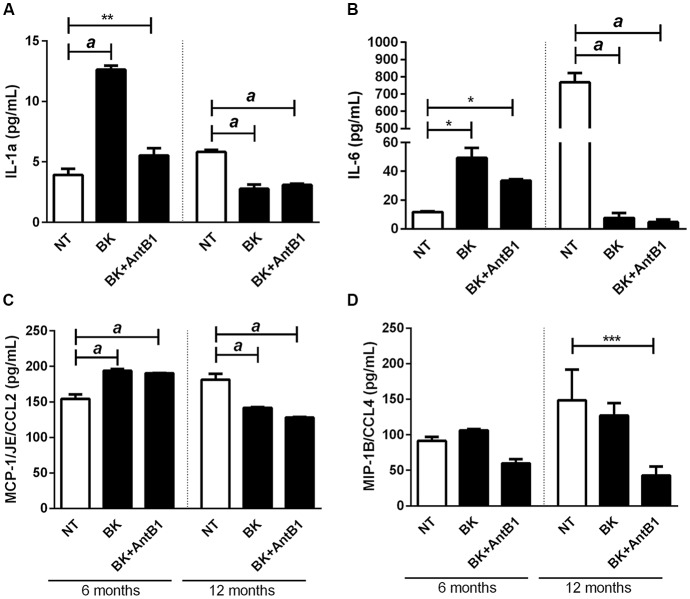
Treatment with bradykinin and B1 antagonist decreases the release of pro-inflammatory cytokines only in 12-OHC. After 30 DIV treatment with 300 pM of BK or 300 pM of BK + 200 pM of B1 antagonist (AntB1) for 30 DIV, the culture medium was collected and the level of cytokines (MCP-1/JE/CCL2, MIP-1B/CCL4, IL-6, IL-1a) evaluated by the Luminex 200 Elisa Multiplex method. **(A)** IL-1a levels; **(B)** IL-6 levels; **(C)** MCP-1/JE/CCL2 levels; **(D)** MIP-1B/CCL4 levels. NT, non-treated slices. Histograms and vertical bars show the means ± SEM. *p < 0.05, **p < 0.01, ***p < 0.001, ^a^*p* < 0.0001, analyzed with one-way ANOVA followed by Bonferroni’s multiple comparisons test. Technical quadruplicate of four animals was used for each treatment.

The same was observed in relation to the other cytokines evaluated, where the level of cytokine release in 6-OHC medium is completely different from the amount released in 12-OHC medium (Figures 5B–D). Moreover, the untreated 12-month-old animals had a higher inflammatory profile than the 6-month-old animals, as shown by IL-6 levels (Figure 5B). These data indicated that the KKS is only effective in reducing the inflammatory profile in middle-aged animals.

### Bradykinin Through the B2 Receptor Confers Protection Against Brain Damage

After verifying that the KKS is involved in lower cell death and decreased the inflammatory profile in adult animals, the next step was to verify if KKS could confer neuroprotection. For this, S100b and BDNF levels were evaluated in 6- and 12-OSHC.

S100b is located in the cytoplasm and nucleus and is involved in the cellular processes regulation such as cell cycle progression, communication, growth, cell division, maintenance of calcium homeostasis, differentiation and energy metabolism regulation. It is expressed primarily in astrocytes and is released into the extracellular space in response to glutamate, serotonin, inflammatory cytokines and amyloid-β peptides (Wang and Bordey, 2008; Zongo et al., 2012). S100b has been used as a brain damage biomarker (Filippidis et al., 2010; Thelin et al., 2016; Park and Hwang, 2018).

**Figure 6:**
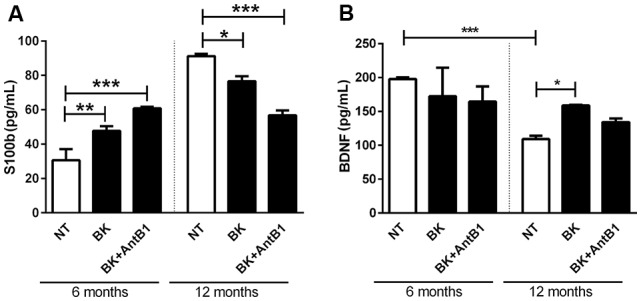
The B2 receptor for bradykinin is important in the neuronal protection process conferred by the kallikrein-kinin system (KKS) system. After 30 DIV of treatment with BK or BK+AntB1, the culture medium was collected and the S100b level **(A)** and brain-derived neurotrophic factor (BDNF; **B**) were analyzed by Elisa. Histograms and vertical bars show the means ± SEM. *p < 0.05, **p < 0.01, ***p < 0.001, analyzed with one-way ANOVA followed by Bonferroni’s multiple comparisons test. Technical triplicate of three animals was used for each treatment.

In samples from 6-OHC treated with BK and BK+AntB1, a greater cell damage profile was observed when compared to those from the control group (55.5%, *p* < 0.01 and 98%, *p* < 0.0001, respectively; Figure 6A). However, in untreated 12-OHC the levels of cell damage are three-fold higher in relation to untreated 6-OHC, as expected. Interestingly, treatments with BK and BK + AntB1 were able to significantly decrease S100b release in 12-OHC (16%, *p* < 0.05 and 37.7% *p* < 0.001; Figure 6A), indicating that the KKS confers a neuroprotective role only in adult animals where a neuroinflammatory process is already present.

BDNF is a member of a small group of neurotrophins that regulate many aspects of neuronal development and function in both the central and peripherical nervous systems. BDNF is responsible for the maintenance of brain function by the induction of survival pathways and neuroplasticity.

As expected, the untreated 6-OHC show higher levels of BDNF than 12-OHC (2-fold; *p* < 0.001; Figure 6B). Bradykinin or Bk+AntB1 do not alter BDNF levels in 6-OHC. However, BDNF levels in 12-OHC treated with BK increased 45.8% (*p* < 0.05; Figure 6B).

## Discussion

The present study demonstrates that OHCs from adult animals can be cultured several weeks while maintaining their viability. Moreover, bradykinin was able to decrease neuroinflammation and increase neuronal plasticity in hippocampal slices from adult animals through activation of its B2 receptor. Over the past two decades, OHCs derived from perinatal animals have been developed, optimized and implemented for a variety of studies (Gähwiler, 1981; Stoppini et al., 1991).

Organotypic brain slice cultures have been used to explore functional development (Finley et al., 2004), brain cells (Franke et al., 2003; Chechneva et al., 2005) and to test new drugs such as neuroprotective substances (Sundstrom et al., 2005; Heine et al., 2013).

Recently, Jang et al. (2018) established organotypic cultures derived from old transgenic animals to study AD, where the slices are grown in serum-free media for 4 weeks. In our work, we established OHCs from 6- and 12-months-old C57Bl/6 animals with serum and demonstrated the ability of bradykinin to maintain viable cultures over a long period.

During the course of cultivation, the slices maintained good morphology with the preservation of hippocampal cytoarchitecture (Figure 1) and low cell death (Figure 2).

KKS has a variety of physiological functions including vasodilatation, proteases activation and inactivation, prostaglandin synthesis stimulation and smooth muscle contractility (Bhoola et al., 1992). Kininogen is the precursor protein of kinins and is cleaved by kininase to generate kallidin and bradykinin (Bhoola et al., 1992; Nitsch et al., 1998). Kinins act through two types of receptors, BKB1R and BKB2R (Marceau et al., 1998; Leeb-Lundberg et al., 2005). Most of the actions of kinins are performed by the B2 receptor, as it has a high affinity for BK and sensitivity to its antagonist HOE-140.

In neurodegeneration, as described previously, BK receptors can modulate the proteolytic process of APP, appearing in different cells expressing these receptors (Nitsch et al., 1998). It has been observed that a single dose of BK applied in the rat hippocampus promotes Tau hyperphosphorylation, leading to decreased learning and memory (Wang and Wang, 2002).

To observe the effect of BKB2R activation on OHC in different ages of mice, we evaluated cell death and neuron markers following BK or BK +AntB1 treatment. Interestingly, the treated slices presented lower cell death when compared to the control slices in both 6-OHC and 12-OHC (Figure 3). However, when NeuN expression was analyzed, the 6-OHC had a lower NeuN density after the treatments and the 12-OHC presented higher neuron density (Figure 4) indicating that, despite the increased general cell viability, the BKB2R activation decreased NeuN protein in 6-OHC. It is important to note that the propidium iodide staining method is widely used to determine cellular viability of OHC but it is not able to discriminate cell types (Happ and Tasker, 2016).

In relation to the 12-OHC, BKB2R activation promoted increase of NeuN protein density, since after the pharmacological inhibition of B1 receptor, the BK action at the B2 receptor exclusively, promoted increase of NeuN and BDNF densities and cellular viability (Figures 3, 4, 6B). These data corroborate the hypothesis that BKB2R activation is effective in neuroprotection only in older animals.

An interesting finding is the decrease of synaptophysin density due to the pharmacological blockade of the BKB1R, suggesting that in some manner this receptor could modulate synapse growth and needs further studies to clarify that.

In agreement with these findings, Martins et al. (2005) demonstrated that BK was important in the formation of embryonic bodies of P19 cells *via* the B2 receptor and in the determination of the cholinergic phenotype of these differentiated neurons, resulting in an autocrine loop for bradykinin release, which was able to induce neurogenesis. Moreover, bradykinin-induced signaling *via* the B2 receptor is essential for the determination of neural fate as well as the specification of neurotransmitter receptor expression in differentiated cells (Martins et al., 2005, 2008).

Lemos et al. (2010) evaluated the role of kinin receptors in memory consolidation during the aging process using wild type (WT), and B1 (KOB1) and B2 (KOB2) knockout mice aged 3, 6, 12 and 18 months. At 12 months of age, the KOB2 animals presented no differences in memory consolidation whereas the WT and KOB1 animals were able to consolidate the memory (Lemos et al., 2010). In a study with human APP transgenic animals, treatment with the B1 antagonist for 10 weeks promoted improved learning and memory (Lacoste et al., 2013). In that study, the blockade of BKB1R may have exacerbated the action of endogenous BK over the BKB2R, similarly to the observed in the present study. In KOB1, KOB2 and WT animals that received chronic infusion of amyloid-β peptide 1–40 in the lateral ventricle at 12 weeks of age and then underwent the active avoidance test, a large decline in memory was observed in the WT and KOB2 animals, but not in the KOB1 animals, suggesting that in the aging process, the B1 receptor may be involved in neurodegeneration and memory loss, while the B2 receptor acts as a neuroprotective factor (Amaral et al., 2010). In addition, after the Aβ chronic infusion, memory processes were evaluated and it was observed that BKB2R genetic deletion accelerated cognitive deficit in comparison to WT animals (Amaral et al., 2010).

It is interesting to note that BK increased BKB1R and BKB2R levels in 6-OHC and the B1 antagonist reduced BKB1R and BKB2R levels (Figure 4). But in 12-OHC, after BKB1R pharmacological inhibition, there was an increase in BKB2R levels (Figure 4), suggesting that *in vivo* there is a possible compensatory effect. In fact, some works demonstrate that in knockout animals some genes that make up certain signaling systems can compensate for the absence of deleted gene by overexpressing other constituents of the system. In the case of KKS, some studies have shown that knockout animals for the B2 receptor compensates this absence by accentuating B1 receptor expression (Austinat et al., 2009). Martins et al. (2012) demonstrated that BKB2R blockade favored the B1 receptor increase. Our results indicate that the BKB2R increase favored by BKB1R antagonism promoted neuroprotection in 12-month-OHC (Figures 3, 4, 6). In addition, it is known that the B1 receptor is expressed at low levels in healthy tissues, as we can see in the untreated slices of the untreated 6-OHC (Figure 4) and B2 receptor expression occurs when a pro-inflammatory stimulus occurs, as occurred with the BK treated 6-OHC (Figure 4).

The role of KKS in neuroinflammation is quite controversial. Although its role in inducing inflammatory cytokines release is already established, some studies have shown that depending on the conditions, KKS may induce a decrease in neuroinflammation. In 3 months old rats it was demonstrated that B2 antagonist injection 2 h before injection of the amyloid-β peptide 1–40 significantly inhibited neuroinflammation, reducing pro-inflammatory protein levels and improving cognitive performance (Bicca et al., 2015). In the same way, in this study, we demonstrated that in 6-OHC, BK treatment increased BKB2R expression (Figure 4) and neuroinflammatory state, while pharmacological inhibition of the B1 receptor resulted in a decrease in inflammatory cytokines (Figure 5). These data are in agreement with the literature, showing that KKS promotes a neuroinflammatory state and BKB2R may contribute to possible neurodegeneration (Viel and Buck, 2011; Bicca et al., 2015).

However, BK treated 12-OHC decreased the release of IL-1α and the pharmacological inhibition of B1 receptor was not able to reverse the release of IL-1α (Figure 5A), demonstrating that BK, through the BKB2R, may have exerted an anti-inflammatory role in middle-aged animals. Further studies using animals treated with BK analogs should be performed to confirm these findings. Regarding CCL2 and CCL4, both BK and the BKB1R antagonist were able to reduce cytokine levels in 12-OHC, showing that in case of CCL2 and CCL4 the B1 receptor acts in combination with the B2 receptor (Figure 5). 12-OHC showed high IL-6 levels that decreased dramatically with BK treatment. The B1 antagonist did not make any difference in the release of IL-6 (Figure 5). It is known that BKB2R is closely related to the inflammatory process; its expression is constitutive and stimulation leads to rapid desensitization leading to increased intracellular Ca^2+^ and pro-inflammatory cytokine release into neurons (Campbell, 2003; Viel and Buck, 2011). On the other hand, the Kinin B1 receptor is mainly expressed in pathological conditions such as chronic inflammation, and has higher affinity to desArg^9^BK and Lys-des-Arg^9^-BK (Viel and Buck, 2011), activating and increasing BKB1R induced MAPK and NF-κB pathway activation (Marceau and Bachvarov, 1998; Marceau et al., 1998; Dias et al., 2015). In other words, depending on the active receptor a specific cytokine profile will be released, which may be happening with our study model.

As previously mentioned, the KKS effect in neuroinflammation is quite controversial. Another study demonstrated the anti-inflammatory role of the B1 receptor both *in vitro* and *in vivo*. In the study, BV2 cells were treated with B1 and B2 agonists, but only the B1 agonist was able to decrease the release of nitric oxide and tumor necrosis factor-alpha (Torika et al., 2014) while the B2 agonist increased COX-2 mRNA and protein levels and prostaglandin E_2_ synthesis in primary rat astrocytes, demonstrating that kinins may exert opposite effects depending on the context.

In the present study, we also demonstrated that kinins exert different effects depending on the context. The12-OHC had a higher inflammatory and toxic profile than 6-OHC (Figures 5, 6). In fact, old age is characterized by a chronic low-grade inflammatory state, with an overexpression of circulating and brain inflammatory factors (Marttila et al., 2011; Mosher and Wyss-Coray, 2014), a phenomenon termed “inflammaging” (Franceschi and Campisi, 2014). This dramatic change in the brain microenvironment, reflected by pro-inflammatory phenotypic changes could facilitate and exacerbate the impairment in synaptic plasticity and cognitive function observed in the aged and AD brain (Udeochu et al., 2016). It was shown that BK can work as an anti-inflammatory or neuroprotective mediator in the brain, through its effect on glial cells increasing the production of prostaglandine E2 and the consequential increase in intracellular cAMP leading to the decrease of TNF-α and IL-1β (Noda et al., 2007). The evaluation of anti-inflammatory cytokines is also relevant to determine the true effects of BK in the “inflammaging,” but even without this data, the decrease in pro-inflammatory cytokines in 12-OHC samples *per se* suggests the participation of BK in the decrease of the inflammatory state on aging. This information is essential for the comprehension of the anti-inflammatory action of kinins in the CNS.

To determinate and control pro- and anti-inflammatory cytokine balance regarding BK action in the CNS may allow us to identify new therapeutic targets for neurological diseases.

In respect of the calcium-binding peptide S100B, used as the parameter of glial activation or death in many diseases of the CNS (Chong et al., 2016), treatment with bradykinin and B1 antagonist was only shown to be beneficial in relation to its neuroprotective role in 12-month-OHC (Figure 6A). In adult animals treatment with bradykinin was detrimental, and moreover, the B2 receptor acting alone was not able to decrease the S100B release (Figure 6A).

These data reinforced the fact that age is a factor responsible for increasing the risks and symptoms of neurodegenerative diseases. Changes in behavior and neurochemistry induced by Aβ are aggravated in aging. The reduction in BDNF is also related to aging. We observed lower BDNF levels in the 12-month-OHC group than in the control group, but bradykinin was able to reverse the decline in BDNF levels (Figure 6B).

## Conclusion

Our data indicated that the KKS may play a neuroprotective role in middle-aged animals, that is, in a context in which the aging process and neuroinflammation is already established.

Also, the organotypic culture from adult and middle-aged animals is an important tool to study neurodegenerative diseases. It allows the study of a greater number of drug combinations, to reduce the number of animals used and better mimic *in vivo* models.

Moreover, we demonstrated that the BKB2R activation by BK increased cell viability of OHC and reduced the pro-inflammatory profile characteristic of neurodegenerative diseases only in old animals, highlighting the importance of BKB2R as a therapeutic target to treat age-related neurodegenerative diseases.

## Data Availability

The raw data supporting the conclusions of this manuscript will be made available by the authors, without undue reservation, to any qualified researcher.

## Ethics Statement

The experimental proceedings were performed according to the ethics principles for the use of laboratory animals of the Brazilian Society of Laboratory Animal Science (SBCAL, Brazil). The experimental protocols were approved by the Animal Ethics Committee from Santa Casa de São Paulo School of Medical Sciences, No. 001/14.

## Author Contributions

MT and HB conceived and designed the experiments. MT, SE and LO performed the experiments. MT, TV and HB analyzed the data and wrote the article. All authors read and approved the final manuscript.

## Conflict of Interest Statement

The authors declare that the research was conducted in the absence of any commercial or financial relationships that could be construed as a potential conflict of interest.
